# Stability and consistency of metamemory judgments within a session

**DOI:** 10.3389/fpsyg.2022.917576

**Published:** 2022-08-22

**Authors:** Michael K. Yeung

**Affiliations:** ^1^Department of Rehabilitation Sciences, The Hong Kong Polytechnic University, Kowloon, Hong Kong SAR, China; ^2^University Research Facility in Behavioral and Systems Neuroscience, The Hong Kong Polytechnic University, Kowloon, Hong Kong SAR, China

**Keywords:** judgment of learning, feeling-of-knowing, retrospective confidence judgments, memory, metacognition, gamma

## Abstract

There has been much interest in assessing individual and group differences in metamemory skills. These endeavors require or would benefit from enhanced knowledge about the stability and consistency of metamemory judgments over successive trials. However, few studies have examined these aspects. Thus, the present study investigated and compared the within-session stability and consistency of three major types of metamemory judgment: judgment of learning (JOL), feeling-of-knowing judgment (FOK), and retrospective confidence judgment (RCJ), using a single-task paradigm. A total of 38 healthy young adults (17 males, 21 females) completed three trials of a face–scene associative learning task designed to assess metamemory. In each trial, participants rated their JOLs while studying a new set of face–scene pairs, and then rated their FOKs and RCJs while their memory was being tested. The stability and consistency of the mean confidence ratings and the relationships between confidence rating and memory performance, indexed by two gamma estimates, were analyzed and compared across types of metamemory judgments. Over trials, there was a significant decrease in the mean rating for JOL but not for FOK or RCJ. Also, the gamma scores of JOL, but not that of FOK or RCJ, significantly improved with practice. Furthermore, for each type of metamemory judgment, the mean confidence rating showed excellent consistency across trials. Depending on the judgment type and gamma estimation method, the consistency of gamma scores ranged from poor to excellent. Thus, the present study clarified the temporal dynamics of various types of metamemory judgments and the consistency of metamemory measures.

## Introduction

Metamemory refers to knowledge about one’s own memory ability, and it involves the interaction between monitoring and control processes (Nelson and Narens, 1990; Koriat and Goldsmith, 1996; Metcalfe, 2000). Metamemory monitoring is believed to play a pivotal role in influencing learning behavior. For example, accurate knowledge about one’s likelihood of retrieving material later and about one’s previous memory performance is crucial for the optimal allocation of study time and the adjustment of study strategies. Monitoring processes occur during different stages of memory, from stimulus encoding to information retrieval. Specifically, at the time of acquisition, individuals can make judgments of learning (JOLs) to predict the likelihood that they can later retrieve the learned information (Schwartz, 1994; Koriat, 1997). At the time of retrieval, people can be asked to make feeling-of-knowing judgments (FOKs), typically but not always solicited after a failure to recall, to anticipate the chance of remembering the information later (Nelson and Narens, 1990; Koriat, 1993; Schwartz, 1994). After performing a memory task, individuals can make retrospective confidence judgments (RCJs) about the accuracy of their answers (Thompson and Mason, 1996; Brewer and Sampaio, 2012).

Conventionally, these metamemory judgments are probed using rating scales and tasks that alternate between the study and the test (Nelson and Narens, 1990). Extensive research has suggested that how people rate their confidence judgments generally correlate with actual memory performance. This is often defined as a positive Goodman–Kruskal (G–K) gamma correlation between confidence rating and memory accuracy (Nelson and Narens, 1990; Thompson and Mason, 1996; Koriat, 1997). That is, within individuals, items that receive a higher JOL or FOK rating are more likely to be remembered later. Also, answers that receive a higher RCJ rating are more likely to have been incorrect than those with a lower rating. This relative accuracy of judgment, which has been of primary interest in metamemory research, was of focus in the present study.

Different theories have been proposed to explain metamemory judgments and accuracy. Brunswik’s (1952) lens model postulates that people use various cues to construct judgments, and hence judgment accuracy is a function of the degree to which the cues being used to construct judgments are predictive of test performance. Similarly, and more recently, Dunlosky and Tauber’s isomechanism framework (2013) posits that all metacognitive judgments are based on the same mechanism. What differs is the cues that are available or activated by the type of timing of the judgment being made, which explains why cue utilization and accuracy differ by judgment type. Indeed, JOL (Koriat and Ma’ayan, 2005; Son and Metcalfe, 2005), FOK (Reder, 1987; Koriat, 1993; Metcalfe et al., 1993; Koriat and Levy-Sadot, 2001), and RCJ (Schwartz, 1994; Metcalfe, 2000) have been shown to rely on various mnemonic cues to predict memory performance, but with different weights. In keeping with this view, studies have demonstrated that the accuracies of different types of metamemory judgments are dissociable or minimally interrelated (Kelemen et al., 2000; Souchay and Isingrini, 2012; Le Berre et al., 2016).

Because metamemory skills are important for efficient learning and memory, much research has been devoted to assessing them across populations, including the aged population (Connor et al., 1997; Perrotin et al., 2006). They have also been assessed with respect to neuropsychiatric disorders associated with memory impairments, including Alzheimer’s disease (Souchay, 2007), schizophrenia (Moritz and Woodward, 2006), and autism spectrum disorder (Grainger et al., 2014). Also, neuropsychological studies comparing healthy individuals and lesion patients have suggested a neural basis of metamemory (Pannu and Kaszniak, 2005; Modirrousta and Fellows, 2008; Chua et al., 2014). These studies require that metamemory measures show sufficient consistency (i.e., reproducibility of the position or rank), at least over a short period of time. Indeed, this characteristic determines the value of any psychological test and forms the basis of drawing meaningful conclusions from data about differences between individuals or groups. In addition, these studies would benefit from enhanced knowledge about the stability of metamemory judgment (i.e., preservation of a score) over the course of the task because it elucidates the temporal dynamics and mechanisms of the judgment (e.g., effects of fatigue and learning).

Unfortunately, few studies have evaluated the stability and consistency of the confidence ratings and the accuracy of metamemory judgments, and some have reported low reliability of metamemory accuracy (i.e., gamma scores) over 1–2 weeks. For example, Kelemen et al. (2000) reported significant test–retest correlations for JOL and FOK ratings but negligible test–retest correlations for JOL and FOK accuracies assessed 1 week apart. Similarly, some studies have shown that across two to three sessions that occurred over at least 1–2 weeks, the accuracy of memory and RCJ ratings exhibited moderate to good reliability, whereas RCJ accuracy possessed very low or at most low-to-moderate reliability (Stankov and Crawford, 1996; Thompson and Mason, 1996; Jonsson and Allwood, 2003).

Indeed, although the evidence has been inconclusive and limited in scope, some studies have shown that metamemory accuracy exhibits systematic changes over trials and it lacks consistency, even within a session. Vesonder and Voss (1985) reported a significant improvement in JOL accuracy from the first to the second word list, such that JOL accuracy was above the chance level only for the second list performance. Nelson and Narens (1990) found significantly improved FOK accuracy over three trials of 15 items, but this finding was not replicated in another experiment with four trials of 10 items. Limited by the dearth of evidence, the stability and consistency of RCJ across trials remains unclear. Also, while a distinction has long been made between JOL, FOK, and RCJ (Nelson and Narens, 1990; Kelemen et al., 2000), the stability and consistency of these different types of metamemory judgments have been mainly examined in isolation using different task paradigms. How these features compare among different types of metamemory judgments remains elusive.

The aim of the present study was to evaluate and compare the within-session stability and consistency of JOL, FOK, and RCJ over three trials of paired-associate learning (i.e., episodic metamemory). Each trial consisted of studying a new set of items, followed by a test of memory. While metamemory is commonly studied with word lists or word pairs, face–scene pairs were used in this study to facilitate the use of this metamemory paradigm for future aging research in Hong Kong. By 2016, 60% of older adults in Hong Kong had attained primary education and below only (Census and Statistics Department, 2018). Thus, using pictorial stimuli, which placed lower demand on literacy than word pairs, would contribute to this research endeavor. In addition, while metamemory accuracy is traditionally indexed by the G–K gamma computed using concordant and discordant pairs of observations, Higham and Higham (2019) recently proposed a new gamma (H–H gamma) that is estimated *via* receiver operating characteristic (ROC) curves and the trapezoidal rule. Simulations have shown that this gamma was more accurate than the traditional gamma in most cases. Thus, both gamma estimates were investigated in this study.

## Materials and methods

### Participants

A total of 51 Chinese adults aged 18–39 years were originally recruited by means of a poster advertisement on the campus of the Hong Kong Polytechnic University. No participant had a history of any psychiatric or neurological disorder or suffered from a stroke or traumatic brain injury that required hospitalization. None was taking any psychotropic medication. All participants abstained from alcohol and caffeine intake on the day of the experiment. Each one self-reported normal or corrected-to-normal vision. Participants provided written informed consent before being tested individually in a quiet room on the university campus. A total of 13 individuals were excluded from subsequent analysis because they had a missing gamma score in at least one test trial (see Section “Data Analysis”). Therefore, the analytic sample consisted of 38 individuals (17 males, 21 females) with a mean age of 25.5 years (*SD* = 5.1).

The sample size was determined based on previous studies addressing the test–retest or split-half reliability of JOLs, FOKs, and/or RCJs. Across studies, the correlations between confidence ratings at two time points ranged from 0.44 to 0.69 (Thompson and Mason, 1996; Kelemen et al., 2000). Assuming a correlation of 0.44, a power of 0.80, and an alpha level of 0.05, the sample size required to detect a significant correlation was 38. Sample size estimation was not based on the correlations between gamma scores at two time points because they have been found to be very small, if not negligible, which led to impractical sample sizes (Thompson and Mason, 1996; Kelemen et al., 2000). This study was approved by the Human Subjects Ethnics Sub-Committee at the Hong Kong Polytechnic University (HSEARS20201110006) and conducted in accordance with the Declaration of Helsinki.

### Test materials

A modified face–scene associative learning task was used to probe metamemory (Chua and Solinger, 2015). Test stimuli included 54 photographs of neutral, front-facing Chinese adult faces taken from the CUHK student database (Wang and Tang, 2008), as well as 54 scenic pictures (18 neutral, 18 positive, and 18 negative) that belonged to the “people” category from the Nencki Affective Picture System (Marchewka et al., 2014). Pictures of different valences were chosen to increase the variability of metamemory ratings and memory performance (Tauber and Dunlosky, 2012). For each individual, the 54 faces and 54 scenes were first randomly paired, and then equally divided into three sets. Each set contained six pictures for each valence. Because there were few pictures for each valence in each trial, and valence had no significant effects on memory performance or metamemory accuracy across items (ANOVA: *p*s > 0.14), valence was excluded from the subsequent analysis. The IDs of the chosen stimuli are given in the Appendix. The task was programmed using E-Prime 3.0 (Psychology Software Tools, Pittsburgh, PA, United States) and administered on a Lenovo 13.3-inch touchscreen laptop.

### Procedure

**Figure 1:**
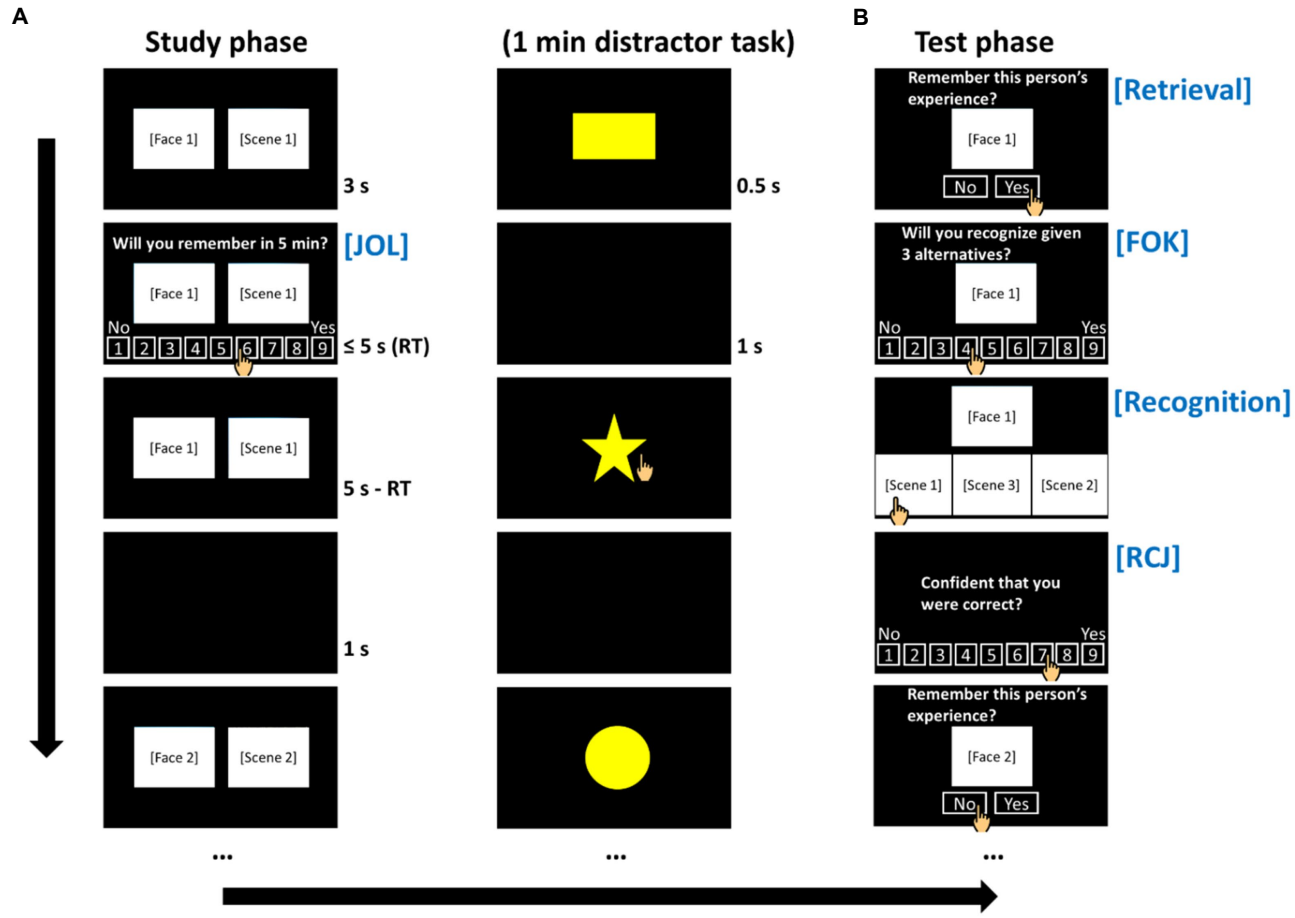
Flow of the metamemory task paradigm. **(A)** The study phase. **(B)** The test phase. FOK, feeling-of-knowing judgment; JOL, judgment of learning; RCJ, retrospective confidence judgment; RT, reaction time. Real-world facial and scenic photographs (not shown) were used in the present study.

The metamemory task consisted of three test trials (Figure 1). Each trial began with a study phase, followed by an intervening task and then a test phase (Chua and Solinger, 2015). During the study phase, pairs of faces and scenes were presented on the left and right sides of a computer screen, respectively, one pair at a time. After a face–scene pair was shown for 3 s, the JOL question (“Will you remember after 5 min?”) along with a Likert scale ranging from 1 (“no”) to 9 (“yes”) also appeared on the screen. Participants made a JOL by touching a scale point within 5 s. The face–scene pair remained onscreen for 5 s, for a total of 8 s of study time per pair, followed by an interstimulus interval of 1 s. This design was based on studies that solicited JOL, regardless of a time limit, while studying the stimulus pairs (Kimball and Metcalfe, 2003; Tullis et al., 2013).

After studying all 18 face–scene pairs, participants performed a visual go/no-go distractor task for 60 s. This duration is in keeping with previous studies, in which 30 s (Leonesio and Nelson, 1990; Logan et al., 2012) or 60 s (Modirrousta and Fellows, 2008) was used. During the distractor task, 10 stars, 10 rectangles, 10 triangles, and 10 circles were presented in a trial-by-trial and a randomized order for each individual. Each shape lasted for 0.5 s, followed by an interstimulus interval of 1 s. Participants touched the screen as quickly as possible whenever a star appeared. This task occurred only once in each trial.

The test phase began after the intervening task. For each item, participants were first shown a previously studied face and prompted to indicate whether they could recall the picture paired with the face (i.e., answering “yes” or “no”). Notably, it is impossible to retrieve every detail of a real-world scenic picture studied only for 8 s; indeed, the “yes” and “no” responses reflected the retrievability of partial rather than complete information (see Section “Retrievability of Information and Its Relationship With Metamemory Judgment”). After that, participants were asked to make a FOK (“Will you recognize given 3 alternatives?”) by rating on a Likert scale again ranging from 1 (“no”) to 9 (“yes”). In keeping with some previous studies, FOKs were solicited regardless of the outcome of the retrieval attempt (Koriat, 1993; Chua et al., 2009; Watier and Collin, 2011; Chua and Solinger, 2015).

After rating the FOK, participants were given a three-alternative forced-choice recognition test, where a face was presented at the top of the screen and three scenic pictures were shown side by side at the bottom (Modirrousta and Fellows, 2008). One picture was the target, while the other two pictures (i.e., foils) were previously paired with either another same-valence face or a different-valence face. Participants chose the picture that paired with the target face by touching the picture. They then made an RCJ (“Confident that you were correct?”) by rating from 1 (“no”) to 9 (“yes”). The next test trial with a different set of stimuli began after all the face–scene pairs had been rated (using the same scale) and tested.

Before the task started for real, participants were briefed on the task instruction and asked to remember the life experiences of different people presented in the form of photographs (left: face; right: scene). They then underwent a study–test practice trial with two stimulus pairs (Leonesio and Nelson, 1990). Thus, the participants knew they would receive a recognition test and what they were supposed to predict. Participants were also informed that they would be answering some rating scale questions as they went along. They were instructed to use the full range of the rating scales.

### Data analysis

Several analyses were conducted to evaluate the stability and consistency of memory performance, metamemory judgment ratings, and metamemory accuracy across trials. Stability (i.e., change over trials) was evaluated using ANOVAs and *t*-tests, and consistency between and among trials was quantified by calculating Pearson’s correlations and intraclass correlation coefficients (ICCs) (Cicchetti, 1994; Koo and Li, 2016), respectively. Memory performance was evaluated by the proportion of correct answers in the recognition test. Metamemory ratings were based on the mean JOL, FOK, and RCJ ratings. Metamemory accuracy was defined as the gamma correlation between the item-by-item JOL, FOK, or RCJ rating and recognition performance, which ranges from −1 (perfect negative relationship) to +1 (perfect positive relationship).

In the present study, gamma was estimated using two methods. First, the G–K gamma, which has been widely used in metamemory research (Nelson and Narens, 1990), is based on the concept of concordant and discordant pairs (Goodman and Kruskal, 1954).^1^ Second, the H–H gamma, which has been recently proposed to be an improved gamma estimate (Higham and Higham, 2019), is computed *via* ROC curves and the trapezoidal rule.^2^ Due to the use of pictorial stimuli and the imperfect relationship between the retrieval response and recognition performance (see Section “Retrievability of Information and Its Relationship With Metamemory Judgment”), FOK accuracy was computed based on all items. While the stability and consistency of the gamma scores were of primary interest, those of the proportion correct and mean ratings also were analyzed to give a context to the gamma scores.

A total of 13 participants had a missing gamma score in at least one trial due to perfect recognition performance or the use of only one rating throughout the entire trial (JOL: *n* = 6; FOK: *n* = 9; RCJ: *n* = 8). Therefore, the analytic sample consisted of 38 participants who provided a complete set of data (i.e., a gamma score for each trial and for each judgment type). Repeated measures ANOVAs were used to assess the stability of the variables. For memory performance, a repeated measures ANOVA with time (trial 1, trial 2, trial 3) as a factor was conducted on the number of correct answers. In addition, two repeated measures ANOVAs with judgment type (JOL, FOK, RCJ) and time as factors were conducted separately on the mean confidence rating and the gamma score. The Greenhouse–Gessier correction was applied when the sphericity assumption was violated.

Pearson’s correlations were calculated to evaluate the consistency of memory performance, metamemory ratings, and metamemory accuracy between trials. Moreover, two-way random effects, consistency ICCs were calculated to evaluate the consistency of the recognition performance, the mean rating, and the gamma scores of each judgment type among all the trials. Consistency is poor for ICCs <0.40, fair for ICCs between 0.40 and 0.59, good for ICCs between 0.60 and 0.74, and excellent for ICCs ≥0.75 (Cicchetti, 1994). Statistical analyses were performed with IBM SPSS Statistics for Windows, Version 26.0 (IBM Corp., Armonk, NY). All statistical tests were two-tailed, and the alpha level was set at 0.05. Holm–Bonferroni correction was used to control for inflated familywise error rates because of multiple comparisons. Uncorrected *p*-values were reported.

## Results

### Memory performance

The means and standard deviations of the primary task variables are presented in Table 1, and the Pearson’s correlations and ICCs of the variables are shown in Table 2. First, the stability and consistency of memory performance across the three test trials were examined. A repeated measures ANOVA with time as a factor showed no significant effect of time for the proportion of correct recognition, *p* = 0.37. After Holm–Bonferroni correction, the proportions of correct recognition were significantly positively correlated among all three trials, *r*s from 0.38 to 0.56, *p*s < 0.018. The single measures ICC was 0.44, and the average measures ICC was 0.71, implying good absolute agreement overall.

**Table 1:** Means and standard deviations of the memory performance, metamemory judgment, and metamemory accuracy variables (*n* = 38).

Variable	Test trials					
	Trial 1		Trial 2		Trial 3	
	*M*	*SD*	*M*	*SD*	*M*	*SD*
**Memory performance**						
Recognition accuracy (%)	71.1	15.0	73.5	13.9	74.4	14.2
**Metamemory judgment**						
Mean JOL rating	5.5	1.6	4.8	1.6	4.6	1.6
Mean FOK rating	5.2	1.4	5.1	1.4	5.1	1.5
Mean RCJ rating	6.1	1.6	6.1	1.7	6.3	1.6
**Metamemory accuracy**						
JOL G–K gamma	−0.11	0.47	0.15	0.46	0.31	0.51
FOK G–K gamma	0.28	0.47	0.38	0.42	0.30	0.51
RCJ G–K gamma	0.43	0.50	0.55	0.42	0.60	0.27
JOL H–H gamma	−0.22	0.43	0.06	0.39	0.19	0.40
FOK H–H gamma	0.14	0.38	0.26	0.30	0.21	0.37
RCJ H–H gamma	0.22	0.50	0.39	0.46	0.34	0.44

FOK, feeling of knowing; G–K, Goodman–Kruskal; H–H, Higham–Higham; JOL, judgment of learning; RCJ, retrospective confidence judgment.

**Table 2:** Pearson’s correlations and intraclass correlation coefficients (ICCS) of the memory performance, metamemory judgment, and metamemory accuracy measures among the three test trials (*n* = 38).

Variable	Pearson’s correlation (*r*)			ICC	
	Trial 1–Trial 2	Trial 2–Trial 3	Trial 1–Trial 3	Single measures	Average measures
**Memory performance**					
Recognition accuracy (%)	0.56***	0.39*	0.38*	0.44 [0.25, 0.63]	0.71 [0.50, 0.84]
**Metamemory judgment**					
Mean JOL rating	0.74***	0.88***	0.64***	0.76 [0.63, 0.85]	0.90 [0.83, 0.95]
Mean FOK rating	0.83***	0.84***	0.81***	0.82 [0.72, 0.90]	0.93 [0.89, 0.96]
Mean RCJ rating	0.78***	0.85***	0.74***	0.79 [0.68, 0.88]	0.92 [0.86, 0.96]
**Metamemory accuracy**					
JOL G–K gamma	0.10	0.34*	0.00	0.15 [−0.04 0.37]	0.34 [−0.13, 0.64]
FOK G–K gamma	0.07	0.18	0.16	0.14 [−0.05, 0.36]	0.33 [−0.15, 0.63]
RCJ G–K gamma	0.06	0.47**	0.23	0.20 [0.00, 0.42]	0.42 [0.01, 0.68]
JOL H–H gamma	0.23	0.42**	0.28	0.30 [0.10, 0.52]	0.57 [0.26, 0.76]
FOK H–H gamma	−0.14	0.19	0.23	0.10 [−0.08, 0.32]	0.25 [−0.28, 0.59]
RCJ H–H gamma	0.57***	0.60***	0.36*	0.50 [0.31, 0.68]	0.75 [0.58, 0.86]

FOK, feeling-of-knowing judgment; G–K, Goodman–Kruskal; H–H, Higham–Higham; JOL, judgment of learning; RCJ, retrospective confidence judgment. The ICCs were based on two-way random effects models with consistency. Bold values survived Holm–Bonferroni correction. *p < 0.05; **p < 0.01; ***p < 0.001.

### Metamemory judgment ratings

**Figure 2:**
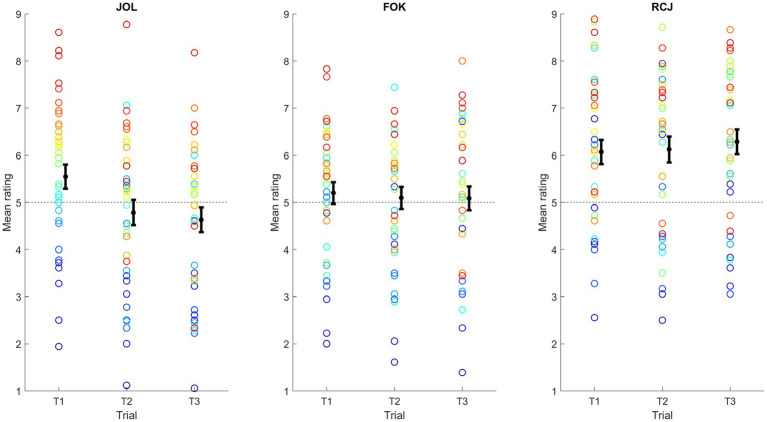
Changes in the mean confidence ratings of metamemory judgments over trials. FOK, feeling-of-knowing judgment; JOL, judgment of learning; RCJ, retrospective confidence judgment. Here, color is used to facilitate comparison of the ranks of individuals across trials and judgment types. Across plots, each individual is represented by the same color, sorted from red to blue by the mean JOL rating in Trial 1. Error bars denote one standard error ± the mean.

Next, the stability and consistency of the ratings of metamemory judgments across the trials were analyzed. For stability (Figure 2), a repeated measures ANOVA with judgment type and time as factors was conducted on the mean rating. The main effect of judgment type was significant, *F*(1.59, 58.91) = 39.30, *p* < 0.001, *η*_p_^2^ = 0.52. This was due to a significantly higher rating for RCJ than for JOL and FOK, *p*s < 0.001. No significant difference in the mean rating between JOL and FOK emerged, *p* = 0.33. Also, the main effect of time was significant, *F*(1.61, 59.43) = 3.97, *p* = 0.032, *η*_p_^2^ = 0.097. This was due to a significant decrease in rating from trial 1 to trial 2, *p* = 0.010, and no significant change thereafter, *p* > 0.99. Importantly, the interaction between judgment type and time was significant, *F*(2.81, 103.95) = 8.13, *p* < 0.001, *η*_p_^2^ = 0.18. Therefore, the effect of time was examined separately for each judgment type. Repeated measures ANOVAs with time as factor revealed a significant result only for JOL, *F*(1.53, 56.49) = 14.35, *p* < 0.001, *η*_p_^2^ = 0.28. The results were not significant for either FOK, *p* = 0.67, or RCJ, *p* = 0.42. For JOL, there was a quadratic decrease in mean rating that diminished over the trials.

**Figure 3:**
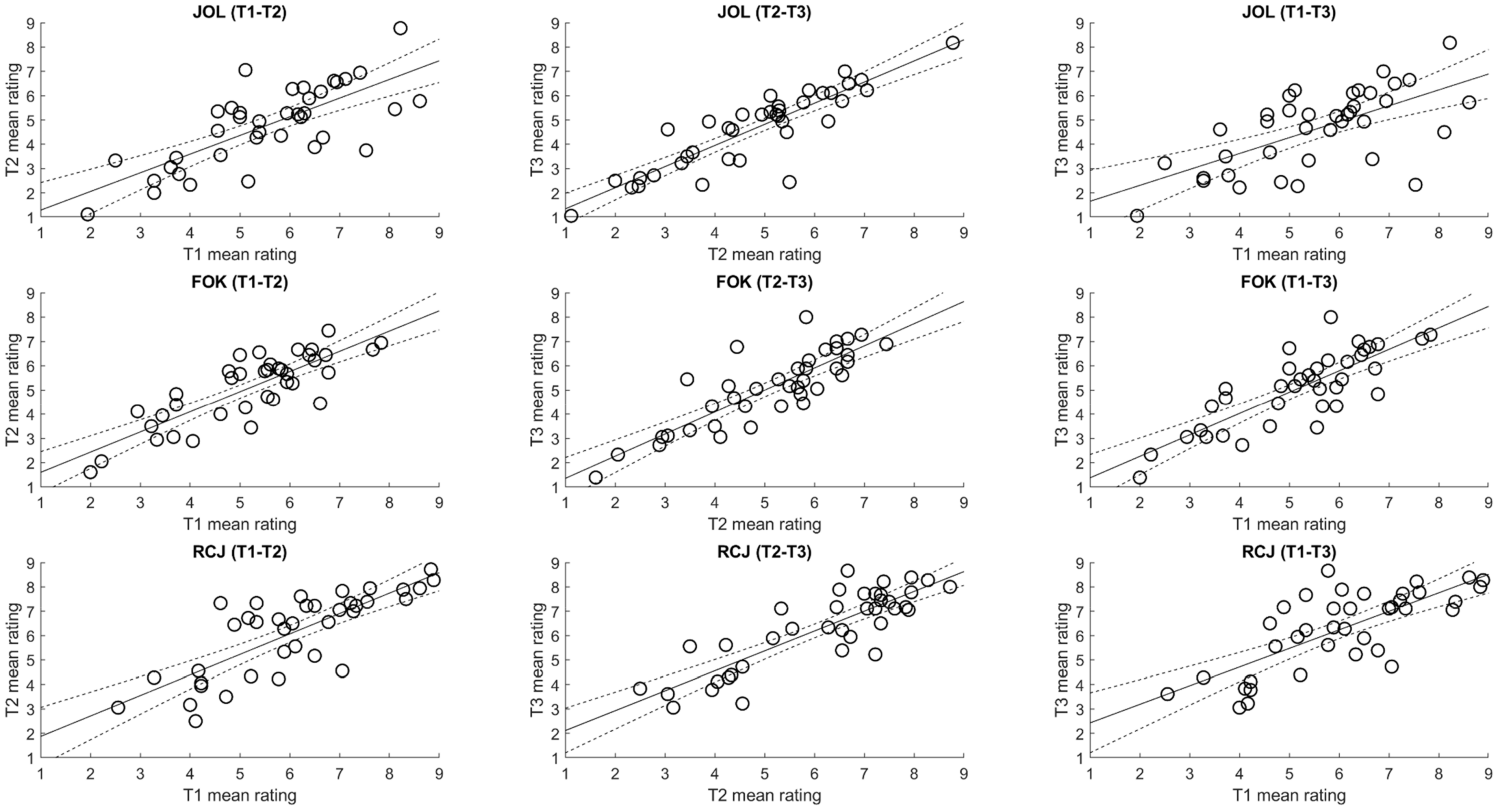
Correlations of the mean confidence ratings of metamemory judgments among trials. FOK, feeling-of-knowing judgment; JOL, judgment of learning; RCJ, retrospective confidence judgment. Dotted lines denote the 95% confidence intervals.

Regarding the consistency of metamemory judgment ratings (Figure 3), after Holm–Bonferroni correction, the mean ratings were significantly positively correlated among all three trials for JOL (*r*s from 0.64 to 0.88, *p*s < 0.001), FOK (*r*s from 0.81 to 0.84, *p*s < 0.001), and RCJ (*r*s from 0.74 to 0.85, *p*s < 0.001). Across different types of metamemory judgments, the single measures ICCs ranged from 0.76 to 0.82, and the average measures ICCs ranged from 0.90 to 0.93. Therefore, the mean ratings exhibited excellent consistency across types of metamemory judgment.

### Metamemory accuracy

**Figure 4:**
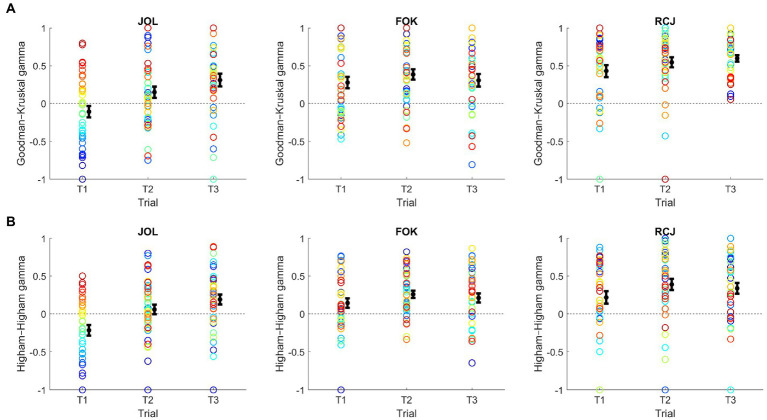
Stability of the **(A)** Goodman–Kruskal and **(B)** Higham–Higham gamma scores over trials. FOK, feeling-of-knowing judgment; JOL, judgment of learning; RCJ, retrospective confidence judgment. Here, color is used to facilitate comparison of the ranks of individuals across trials and judgment types. Across plots, each individual is represented by the same color, sorted from red to blue by the JOL gamma score in Trial 1. Error bars denote one standard error ± the mean.

The stability and consistency of metamemory accuracy across trials were then analyzed. Regarding stability (Figure 4), a repeated measures ANOVA with judgment type and time as factors conducted on the G–K gamma score showed a significant main effect of judgment type, *F*(2, 74) = 19.14, *p* < 0.001, *η*_p_^2^ = 0.34. This was due to a significantly larger gamma score for RCJ than for JOL and FOK, and for FOK than for JOL, *p*s < 0.003. There was also a significant main effect of time, *F*(2, 74) = 5.42, *p* = 0.006, *η*_p_^2^ = 0.13. This was owing to a significant linear increase in the gamma score over trials, *p* = 0.004. Importantly, the interaction between judgment type and time also was significant, *F*(4, 148) = 2.77, *p* = 0.030, *η*_p_^2^ = 0.070. Hence, the effect of time was analyzed by conducting a repeated measures ANOVA with time as a factor for each judgment type.

The repeated measures ANOVAs identified a significant main effect of time only for JOL, *F*(2, 74) = 8.49, *p* < 0.001, *η*_p_^2^ = 0.19. It was not significant for either FOK, *p* = 0.54, or RCJ, *p* = 0.14. This result was attributable to a significant linear increase in the JOL gamma score over trials, *p* < 0.001. Indeed, after Holm–Bonferroni correction, one-sample *t*-tests showed that all but two gamma scores were significantly larger than zero and that the JOL gamma score did not significantly predict recognition performance until the third trial (Trial 1 JOL: *p* = 0.16; Trial 2 JOL: *p* = 0.054; Others: *p*s < 0.001).

Albeit with lower magnitudes overall, the H–H gamma scores yielded almost the same ANOVA and *t*-test results. That is, ANOVA still revealed significant main effects of judgment type, *F*(1.6, 59.7) = 10.79, *p* < 0.001, *η*_p_^2^ = 0.23, and time, *F*(2, 74) = 8.85, *p* < 0.001, *η*_p_^2^ = 0.19. There also was a significant interaction between judgment type and time, *F*(4, 148) = 3.69, *p* = 0.007, *η*_p_^2^ = 0.09, which was driven by a significant linear increase over trials for JOL, *p* < 0.001, but not for others, *p*s > 0.17. Most H–H gamma scores, particularly those representing the last trial, *p*s < 0.006, were significantly above zero.

**Figure 5:**
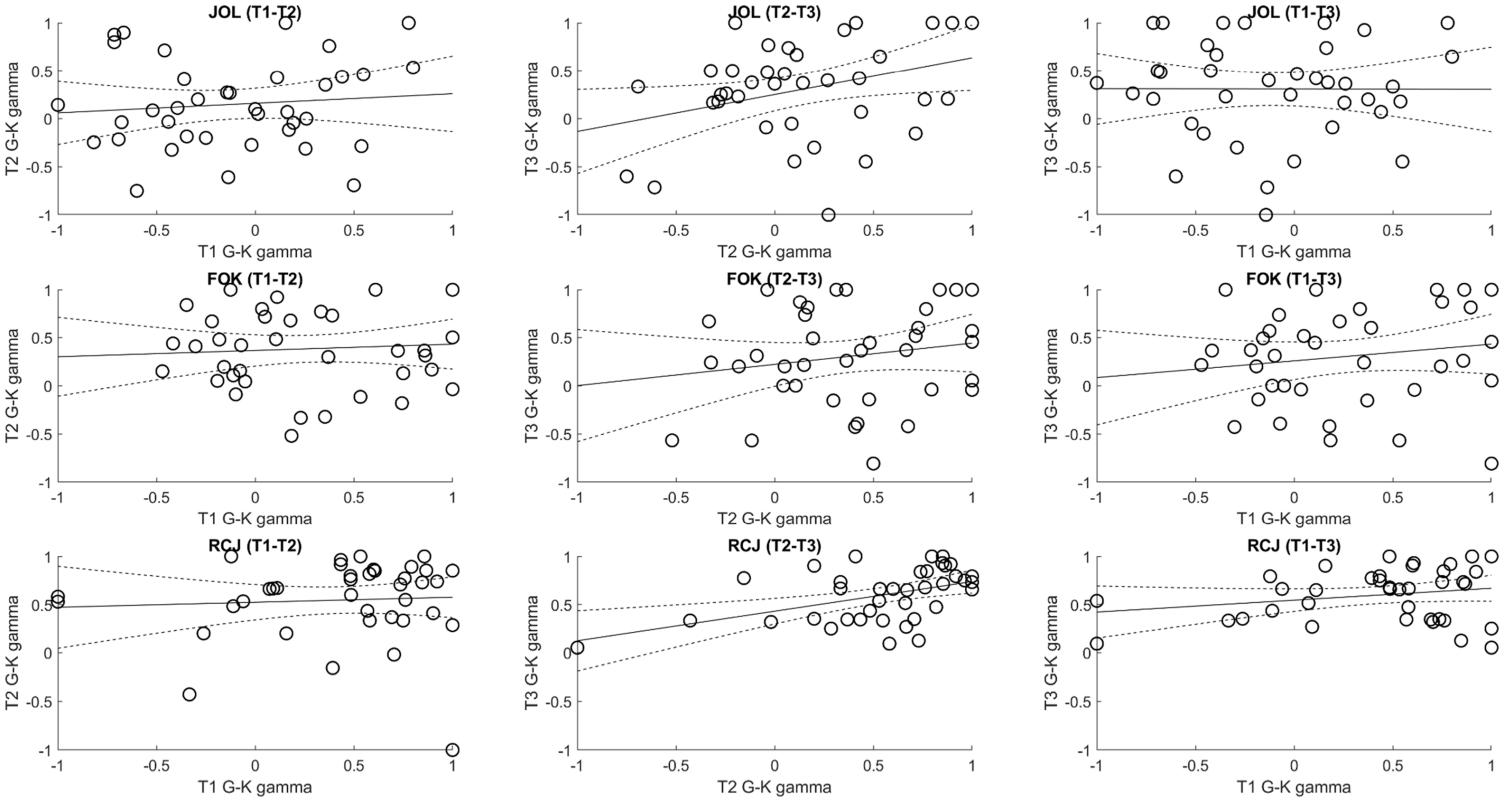
Correlations of the Goodman–Kruskal (G–K) gamma scores among trials. FOK, feeling-of-knowing judgment; G–K, Goodman–Kruskal; JOL, judgment of learning; RCJ, retrospective confidence judgment. Dotted lines denote the 95% confidence intervals.

Regarding the consistency of gamma scores, the G–K gamma scores generally showed poor consistency over the trials (Figure 5). Specifically, after Holm–Bonferroni correction, there was only a marginally significant positive correlation between trial 2 and trial 3 JOL gamma scores, *r*(36) = 0.34, *p* = 0.034, and a significant positive correlation between trial 2 and trial 3 RCJ gamma scores, *r*(36) = 0.47, *p* = 0.003 (others: *p*s from −0.003 to 0.23, *p*s > 0.17). Across types of metamemory judgment, the average measures ICCs ranged from 0.30 to 0.42, implying poor consistency overall.

**Figure 6:**
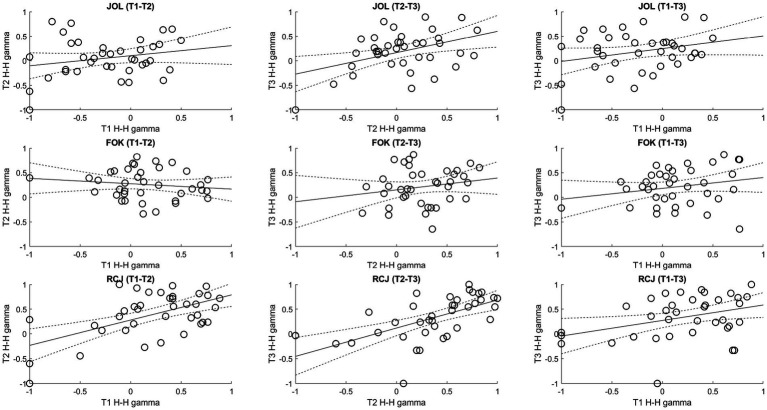
Correlations of the Higham–Higham (H–H) gamma scores among trials. FOK, feeling-of-knowing judgment; H–H, Higham–Higham; JOL, judgment of learning; RCJ, retrospective confidence judgment. Dotted lines denote the 95% confidence intervals.

In contrast, the consistency of H–H gamma scores varied greatly across different types of metamemory judgment (Figure 6). The correlation between trial 2 and trial 3 JOL gamma scores and the correlations among the three trials’ RCJ gamma scores were (marginally) significant, *r*s > 0.36, *p*s < 0.028. In addition, the single measures ranged from 0.10 to 0.50, and the average measures ICCs for JOL, FOK, and RCJ were 0.57, 0.25, and 0.75, suggesting fair-to-good, poor, and good-to-excellent consistency, respectively.

### Retrievability of information and its relationship with metamemory judgment

Additional analyses were performed to clarify the mechanisms underlying the metamemory processes, and the results are shown in Table 3. Before making a FOK, the participants were prompted to indicate whether they could retrieve the scenic picture associated with the face (i.e., making a retrieval attempt). To clarify the nature of the retrieved information, the mean FOK ratings and the proportions of correct recognition following a “yes” and a “no” response were compared by conducting two separate repeated measures ANOVAs with retrievability (yes, no) and time as factors. Unfortunately, only 24 (10 males, 14 females) out of the 38 participants allowed for this analysis, as they gave a mix of “yes” and “no” responses on each of the three trials, with no significant difference in the proportion of items that received a “yes” answer among trials, *p* = 0.46. Nevertheless, comparing those who were subsequently included and excluded did not reveal a significant difference in any variable, including the proportion of items with a “yes” response, *p* > 0.16. Also, none of the previously reported results significantly changed after repeating the analyses (e.g., ANOVAs) with this subset of the sample. Thus, these 24 individuals appear to be representative of the entire study sample.

**Table 3:** Means and standard deviations of variables related to the retrieval response, nature of the retrieved information, and the relationship between the ratings and retrieval responses (*n* = 24).

Variable	Test trials					
	Trial 1		Trial 2		Trial 3	
	*M*	*SD*	*M*	*SD*	*M*	*SD*
**Retrieval response**						
Proportion of answering “yes” (%)	47.1	33.2	47.1	33.9	44.3	33.0
**Nature of the retrieved information**						
Mean FOK rating after answering “yes”	6.8	1.3	6.7	1.3	7.0	1.0
Mean FOK rating after answering “no”	4.3	1.3	4.1	1.3	3.8	1.4
Recognition accuracy after answering “yes” (%)	81.5	18.3	86.3	17.8	83.6	17.9
Recognition accuracy after answering “no” (%)	70.0	18.2	73.4	15.1	65.6	20.2
**Relationship between the ratings and retrieval responses**						
JOL G–K gamma	0.09	0.51	0.29	0.36	0.37	0.44
FOK G–K gamma	0.77	0.44	0.80	0.27	0.96	0.09
RCJ G–K gamma	0.58	0.35	0.63	0.29	0.69	0.32
JOL H–H gamma	0.00	0.46	0.21	0.34	0.31	0.41
FOK H–H gamma	0.73	0.30	0.73	0.29	0.88	0.15
RCJ H–H gamma	0.32	0.47	0.41	0.40	0.39	0.40

FOK, feeling of knowing; G–K, Goodman–Kruskal; H–H, Higham–Higham; JOL, judgment of learning; RCJ, retrospective confidence judgment.

For the mean FOK rating, a significant result was obtained for the main effect of retrievability, *F*(1, 23) = 108.64, *p* < 0.001, *η*_p_^2^ = 0.83. However, the main effect of time was not significant, *p* = 0.44. The interaction between retrievability and time was marginally significant, *F*(2, 46) = 3.18, *p* = 0.051, *η*_p_^2^ = 0.12. *Post-hoc t*-tests revealed a significant difference between the “yes” and “no” responses on each trial, *p*s < 0.001. The interaction was due to a significant linear increase in the difference in mean FOK rating between the two responses over trials, *p* = 0.030. In addition, for the proportion of correct recognition, the main effect of retrievability was significant, *F*(1, 23) = 22.33, *p* < 0.001, *η*_p_^2^ = 0.49. This owed to a more accurate recognition performance following the “yes” than the “no” response. No other effect was significant, *p*s > 0.20. Notably, the mean FOK ratings and recognition performances following the “yes” and “no” responses were far from extreme, suggesting that the two responses likely reflected the retrieval of different amounts of partial information rather than of the complete presence and absence of information.

To elucidate whether the rating of metamemory judgment correlated with the amount of retrieved information cued by a face, a repeated measures ANOVA with judgment type and time as factors was conducted on the G–K gamma correlation between the item-by-item rating and retrievability. The main effect of time was significant, *F*(2, 46) = 4.82, *p* = 0.013, *η*_p_^2^ = 0.17. This could be understood as a significant linear increase in the gamma score over trials, *p* = 0.014. The main effect of the judgment type also was significant, *F*(2, 46) = 53.67, *p* < 0.001, *η*_p_^2^ = 0.70. This was driven by a larger FOK gamma score than the other two gamma scores, and by a larger RCJ gamma score than the JOL gamma score, *p*s < 0.001. No significant interaction between judgment type and time emerged, *p* = 0.52. Using the H–H gamma gave similar results (Time: *p* = 0.018; Judgment Type: *p* < 0.001; Time × Judgment Type: *p* = 0.34).

## Discussion

Metamemory is important for selecting and applying learning strategies that promote memory (Nelson and Narens, 1990). Therefore, there has been much interest in studying and assessing metamemory skills across healthy and clinical populations (Connor et al., 1997; Perrotin et al., 2006; Souchay, 2007; Grainger et al., 2014). The assessment of metamemory relies on the premise that measures of metamemory skills are consistent across repeated measurements; otherwise, these measures cannot be used to infer the true ability of a person. Also, the stability or change of metamemory judgment over the course of the task is important to consider since it informs the temporal dynamics and mechanisms of the judgment. Unfortunately, these aspects have rarely been systematically or comprehensively evaluated. The aim of the present study was to bridge this knowledge gap by comparing the within-session stability and consistency of three major types of metamemory judgments using a single-task paradigm.

The present study focused on gamma (relative accuracy) that was estimated using two methods: the traditional method that considers concordant and discordant pairs (i.e., G–K gamma; Goodman and Kruskal, 1954), and a recent method that is based on area under the ROC curve (i.e., H–H gamma; Higham and Higham, 2019). Regardless of the measure, there was a significant improvement in JOL accuracy but not in FOK or RCJ accuracy over the trials. This selective change was qualified by a significant interaction between judgment type and time. This finding corroborates a previous finding of a significant increase in JOL accuracy from the first to the second trial of list learning, such that JOL ratings did not significantly predict memory performance until the latter stage of the task (Vesonder and Voss, 1985).

In addition, the current finding is consistent with the inconclusive change in FOK accuracy within sessions. Specifically, Nelson and Narens (1990) found that FOK accuracy gradually improved over three test trials in one experiment, but this finding was not replicated in another experiment. To the best of my knowledge, no study has examined the stability of RCJ accuracy within sessions. Nevertheless, like the present study, one study reported no significant change in RCJ accuracy over 2 weeks (Jonsson and Allwood, 2003). Taken together, the present study yields novel evidence that among the three major types of metamemory judgment, only JOL accuracy exhibits systematic changes over successive trials.

According to the isomechanism theory (Dunlosky and Tauber, 2013), all metacognitive judgments are based on the same mechanism. However, since the availability and activation of cues differ by the type of timing of the judgment being made, cue utilization and accuracy differ by judgment type. In the present study, the decrease in JOL ratings over trials suggests that the participants might be adjusting their expectations of their own memory performance based on their performance on prior memory tests (Finn and Metcalfe, 2007). In addition, the improved JOL accuracy over time may imply that the participants learned something about the task and applied it to changing how they made their JOLs going forward. Because a significant improvement in accuracy was observed only for JOL, the adjustment and learning appear to be specific to cue utilization at the time of studying. For example, valence could be an obvious cue during JOL (Tauber and Dunlosky, 2012), but it did not predict memory performance in the present study. The participants might change from relying on valence to other (diagnostic) cues when making JOLs over the course of the task.

Recognition performance and mean confidence ratings were found to exhibit good to excellent consistency across types of metamemory judgment. The H–H gamma scores for JOL and RCJ also demonstrated at least fair-to-good consistency. Thus, although the gamma scores were defined as the item-by-item relationships between confidence ratings and memory performance, the poor consistency of some gamma scores, particularly FOK gamma scores, cannot be simply attributable to unreliable confidence ratings or memory performance. In addition, the relatively small number of items (i.e., 18) per trial, although still larger than that in some studies (Nelson and Narens, 1990; Pinon et al., 2005; Logan et al., 2012), is unlikely to explain the inconsistency in these scores.

Indeed, the consistency of judgment accuracy was found to vary by the type of metamemory judgment and by the gamma estimation method. For JOL and RCJ, the H–H gamma showed improved consistency compared to the G–K gamma. For FOK, however, neither gamma measure yielded consistent results. Higham and Higham (2019) performed a series of simulations and found that the ROC-based gamma deviated less from the true value of gamma than did the traditional gamma in most of the simulations (Higham and Higham, 2019). Therefore, the higher accuracy of the ROC-based gamma may contribute to the enhanced consistency of this new measure, at least for JOL and RCJ. In addition, based on various theories of metacognitive judgment (Brunswik, 1952; Dunlosky and Tauber, 2013), the observed wide range of consistencies across the new gamma scores may indicate that cue utilization undergoes varying extents of idiosyncratic trial-by-trial fluctuation depending on the stage of judgment about learning and memory.

Metamemory judgments are well known to be based on a combination of intrinsic, extrinsic, and mnemonic cues (Koriat, 1997; Koriat and Levy-Sadot, 2001; Koriat and Ma’ayan, 2005; Son and Metcalfe, 2005). Koriat (1997) showed that JOL accuracy improved with practice, and the improved resolution was associated with an increased reliance on mnemonic–experiential cues rather than on intrinsic cues. The present task involved the random pairing of faces and scenes that were different for each individual. Therefore, a change in the reliance on intrinsic cues, such as item relatedness, is unlikely to underlie the selective improvement in JOL accuracy over trials.

The item-by-item correlation between confidence ratings and the retrievability of information significantly increased over trials. This implies an increased reliance on the accessibility of information at the time of judgment. However, this increased correlation was not significantly moderated by the type of judgment. Therefore, how the increase in using this mnemonic cue is linked with the specific improvement in JOL accuracy over trials remains elusive. In addition, as in most metamemory studies, the time interval between metamemory judgment and memory performance was longest for JOL. Therefore, an enhanced use of extrinsic cues (e.g., task structure) that became increasingly apparent over practice, as well as a more accurate estimation of the time interval between the study and the test, may have the greatest impact on JOL accuracy.

In some studies, the G–K gamma scores exhibited poor test–retest reliability over 1–2 weeks. Kelemen et al. (2000) reported significant correlations for JOL and FOK ratings but not for the corresponding gamma scores between two sessions that were 1 week apart. Also, Thompson and Mason (1996) found that the levels of split-half and alternate-forms reliability were moderate-to-good for memory performance and median confidence ratings. However, they were poor for RCJ accuracy assessed on two occasions that took place over a two-week interval (also see Stankov and Crawford, 1996; Jonsson and Allwood, 2003). The present study addressed the within-session stability and consistency of metamemory judgments, and the findings suggest that the poor reliability of these gamma scores can also be detected within one test session. Considering the improved within-session consistency of the ROC-based gamma, future work would benefit from evaluating the reliability of this new measure as applied to evaluating the accuracy of metamemory judgments across sessions.

The present study has several notable strengths. First, it employed a within-subjects design and compared three major types of metamemory judgments using a single, ecologically valid task paradigm. This approach allowed for the direct comparison of different kinds of metamemory judgments with minimal confounds. Second, this study was one of the first to systematically evaluate the within-session stability and reliability of different metamemory variables. It contributes to the literature by revealing the temporal dynamics of metamemory judgments and by clarifying the psychometric properties of several commonly used metamemory metrics. Third, it pioneered to evaluate and compare the stability and consistency of two different gamma estimates. In light of the current findings, the ROC-based gamma is recommended over the traditional gamma for evaluating the relative accuracy of metamemory judgments, particularly JOL and RCJ.

Notwithstanding these contributions, this study has some limitations. First, it addressed only the monitoring aspect of metamemory. The stability and consistency of the control processes of metamemory, including the allocation of study time and the selection of search strategies, remain unclear. Second, ease-of-learning judgments, which have been considered a core monitoring process of metamemory that takes place prior to acquisition (Nelson and Narens, 1990), were not examined. Third, this study did not provide information about the stability and consistency of metamemory judgment ratings and accuracies over repeated exposure to the same set of study items (Koriat, 1997). Fourth, the present results were based on healthy young adults, and they might not be generalizable to other age groups or clinical populations.

In summary, this study examined and compared the within-session stability and consistency of three major types of metamemory judgments using a single-task paradigm. The results showed that only JOL accuracy improved over trials, and this observation was paralleled by a selective decrease in mean JOL ratings. Depending on the type of metamemory judgment and the choice of gamma estimate, the within-session consistency of metamemory accuracy varied greatly, ranging from poor to excellent. This study provides preliminary support for using the ROC-based gamma over the traditional gamma to evaluate the relative accuracy of judgment. It also highlights the need for more research on the temporal dynamics of metamemory judgment and on the psychometric properties of metamemory accuracy measures to improve the understanding and assessment of metamemory abilities.

## Data availability statement

The raw data supporting the conclusions of this article will be made available by the authors, without undue reservation.

## Ethics statement

The studies involving human participants were reviewed and approved by The Human Subjects Ethnics Sub-Committee at the Hong Kong Polytechnic University (HSEARS20201110006). The patients/participants provided their written informed consent to participate in this study.

## Author contributions

MY contributed to study conception and design, data analysis and interpretation, and manuscript writing.

## Funding

This study was supported by the Start-up Fund for RAPs under the Strategic Hiring Scheme (P0034754) and by the One-Line Budget (P0039302) awarded by the Hong Kong Polytechnic University to MY.

## Conflict of interest

The author declares that the research was conducted in the absence of any commercial or financial relationships that could be construed as a potential conflict of interest.

## Publisher’s note

All claims expressed in this article are solely those of the authors and do not necessarily represent those of their affiliated organizations, or those of the publisher, the editors and the reviewers. Any product that may be evaluated in this article, or claim that may be made by its manufacturer, is not guaranteed or endorsed by the publisher.

## Footnotes

^1^G–K gamma = $\left(C-D\right)/\left(C+D\right)$, where *C* and *D* refer to the numbers of concordant and discordant pairs, respectively.

^2^H–H gamma = $\overset{n}{\underset{k=0}{\sum }}\left(HR_{k+1}+HR_{k}\right)\left(FAR_{k+1}-FAR_{k}\right)$, where HR and FAR represent the hit rate and the false alarm rate, respectively; *k* represents the different criteria plotted on the ROC, and *n* refers to the number of criteria.
