# Supplementary material for: Stability and consistency of metamemory judgments within a session
Source: Front Psychol. 2022 Aug 22;13:917576. doi: 10.3389/fpsyg.2022.917576 (PMC9443848; doi:10.3389/fpsyg.2022.917576)
Supplement: Supplementary file 1 [file Table_1.DOCX]

**Appendix: IDs of the Test Stimuli**

**Facial Stimuli from the CUHK Student Database (Wang & Tang, 2009)**

f-006-01, f-009-01, f-011-01, f-012-01, f-013-01, f-014-01, f-015-01, f-017-01, f-019-01, f-023-01, f-026-01, f-027-01, f-033-01, f-034-01, f-035-01, f-039-01, f-043-01, f1-001-01, f1-003-01, f1-004-01, f1-007-01, f1-008-01, f1-009-01, f1-010-01, f1-011-01, f1-012-01, f1-013-01, m-009-01, m-011-01, m-012-01, m-013-01, m-015-01, m-019-01, m-022-01, m-023-01, m-024-01, m-030-01, m-041-01, m-047-01, m-051-01, m-052-01, m-053-01, m-071-01, m-077-01, m1-001-01, m1-016-01, m1-018-01, m1-021-01, m1-023-01, m1-024-01, m1-030-01, m1-031-01, m1-035-01, m1-036-01

**Scenic Pictures from the Nencki Affective Picture System (Marchewka et al., 2014)**

People_020_h, People_022_h, People_026_h, People_028_h, People_035_h, People_036_h, People_038_h, People_042_h, People_043_h, People_051_h, People_052_h, People_054_h, People_055_h, People_061_h, People_065_h, People_091_h, People_095_h, People_096_h, People_097_h, People_100_h, People_103_h, People_104_h, People_110_h, People_113_h, People_115_h, People_116_h, People_122_h, People_127_h, People_128_h, People_140_h, People_146_h, People_149_h, People_150_h, People_155_h, People_159_h, People_164_h, People_169_h, People_185_h, People_186_h, People_187_h, People_190_h, People_196_h, People_198_h, People_200_h, People_208_h, People_221_h, People_222_h, People_226_h, People_227_h, People_237_h, People_238_h, People_239_h, People_240_h, People_246_h,

**References**

Marchewka, A., Żurawski, Ł., Jednoróg, K., & Grabowska, A. (2014). The Nencki Affective Picture System (NAPS): Introduction to a novel, standardized, wide-range, high-quality, realistic picture database. *Behavior research methods*, *46*(2), 596-610. https://doi.org/10.3758/s13428-013-0379-1

Wang, X., & Tang, X. (2008). Face photo-sketch synthesis and recognition. *IEEE transactions on pattern analysis and machine intelligence*, *31*(11), 1955-1967. https://doi.org/10.1109/TPAMI.2008.222
